# Exploring the Performance Advantages of p-Aminobenzenesulfonate-Based Zwitterionic Gemini Surfactants in Oil Recovery

**DOI:** 10.3390/molecules30071537

**Published:** 2025-03-30

**Authors:** Zhaozheng Song, Shiyuan Xia, Tongji Yang, Zhihong Li, Jiayi Li

**Affiliations:** 1 College of Engineering, China University of Petroleum-Beijing, Karamay 834000, China; 2 College of Science, China University of Petroleum-Beijing, Beijing 102249, China; 15928933451@139.com (T.Y.); lizhihong0304@163.com (Z.L.); yi66737yi@163.com (J.L.)

**Keywords:** sodium sulfanilate, zwitterionic gemini surfactant, enhanced oil recovery, interfacial tension

## Abstract

To investigate the specific performance enhancement of oilfield surfactants by using sodium p-aminobenzenesulfonate as a connecting group, cationic surfactant N,N-dimethyl-N-(oxiran-2-ylmethyl)dodecan-1-aminium (DDPA) and zwitterionic gemini surfactant sodium 4-[bis(3-(dodecyldimethylamino)-2-hydroxypropyl)amino]benzenesulfonate (DDBS) were synthesized. The oil recovery performance of these surfactants was compared, revealing that DDBS outperforms DDPA in thermal stability, wettability, adsorption, and resistance to temperature and salinity variations, as well as surface/interface activity, except for emulsification. Core flooding experiments, simulating the conditions of the Xinjiang oilfield, demonstrated that DDBS can achieve the same enhanced oil recovery effect at a concentration that is 1/15 of that of DDPA. Compared with water, DDBS and DDPA can incrementally enhance recovery rates by 7.9% and 8.5%. Furthermore, the synergistic formulation of DDBS with sodium dodecylbenzenesulfonate (SDS) significantly optimized performance, achieving a reduction in interfacial tension to 0.0301 mN m^−1^. This study provides a research and data foundation for the application of new surfactants in petroleum extraction.

## 1. Introduction

Amidst the complex and evolving international landscape, the security of the petroleum industry has garnered significant attention due to its critical importance in mature economies. Despite the fact that primary and secondary oil recovery methods have been in use for over a century and a half, a considerable portion—nearly two-thirds—of the original oil in place (OOIP) remains unrecoverable in many developed oil fields [1,2,3,4]. To enhance oil recovery rates, “tertiary oil recovery” has emerged as a novel and crucial method within the realm of enhanced oil recovery (EOR). This technique typically involves the injection of specially formulated chemicals into the reservoir to modify the properties of the reservoir fluids [5,6]. Among the tertiary oil recovery technologies, the surfactant-based ternary composite flooding technique has garnered considerable attention due to its high development potential and advanced research. It has demonstrated remarkable experimental outcomes in major oil fields such as Xinjiang, Daqing, and Shengli [7,8]. Surfactants can effectively enhance oil recovery through multiple mechanisms, such as reducing oil–water interfacial tension, reversing rock surface wettability, emulsification, and aggregating oil droplets into oil bands. Among these, zwitterionic surfactants and gemini surfactants are notable for their high efficiency in improving oil recovery and their adaptability to the harsh reservoir conditions characterized by high temperature and high concentrations of Na^+^, Mg^2+^, and Ca^2+^ [9,10].

Zwitterionic surfactants, characterized by the presence of both cationic and anionic hydrophilic groups within their molecular structure, exhibit the unique ability to both accept and donate protons. This dual functionality endows these agents with superior dispersibility, mold inhibition, salt resistance, emulsification capabilities, and thermal stability [11,12]. Compared to other surfactant classes, amphoteric surfactants demonstrate reduced sensitivity to temperature fluctuations, pH variations, and the presence of inorganic electrolytes. Their expanded hydrophilic head group results in a lower critical micelle concentration (cmc) and a reduced operational concentration [13,14]. These attributes synergistically augment their versatility and broad-spectrum applicability within the realm of oil recovery.

Gemini surfactants represent a class of innovative specialty surfactants, consisting of two or more single-molecule surfactants bridged by specific linking groups. The molecular architecture of gemini surfactants is distinguished by the presence of two sets of hydrophobic and hydrophilic groups, which adeptly modulate intra-molecular interactions. The incorporation of linking groups not only mitigates electrostatic repulsion among polar groups but also amplifies the hydrophobic interactions of non-polar groups. This refined molecular configuration results in a more condensed aggregation at interfaces, leading to superior interfacial tension reduction [15,16,17,18]. Gemini surfactants exhibit exceptional temperature and salt resistance, along with enhanced wetting properties. They are characterized by a lower cmc and Krafft point, enabling their effective operation across a broader spectrum of temperature and salinity conditions [19,20,21].

Zwitterionic gemini surfactants can be classified based on their hydrophilic group types into amino acid-based, phosphate-based, and betaine-based surfactants. Amino acid-based surfactants exhibit variable characteristics across different pH values [22], with low toxicity and irritation [23] but limited thermal and salt tolerance, making them suitable for mild, pH-responsive applications. Phosphate-based surfactants offer superior interfacial activity and salt tolerance [24]; however, their synthesis involves hazardous chemicals, complicates by-product treatment, and poses higher ecotoxicity risks [25]. Betaine-based surfactants, despite the higher raw material and process costs due to the introduction of quaternary ammonium groups, possess excellent wetting properties, interfacial activity, thermal and salt tolerance, emulsification ability, and low adsorption values. Although their cost is relatively high, these properties make them an optimal choice for high-temperature, high-salinity oil recovery operations [26,27,28]. Sodium sulfonamide can serve as a coupling agent in Gemini surfactants, and its sulfonic acid group can also introduce anions into cationic surfactants, thereby forming zwitterionic gemini surfactants. However, the benzene ring in sodium sulfonamide makes it hydrophobic, and it is part of the hydrophilic chain segment, which makes it complex to predict its performance enhancement on single-chain cationic surfactants. Comprehensive and systematic testing is needed to evaluate these impacts, making it a significant experiment.

Tsukasa et al. [29] conducted a study to elucidate the impact of the structure and type of linking groups on the adsorption kinetics of gemini and trimeric surfactants. The authors employed the maximum pressure bubble method to measure the dynamic interfacial tension and utilized the Rosen adsorption model to fit and analyze the diffusion control model. The findings revealed that gemini and trimeric surfactants with highly flexible linking groups exhibit enhanced adsorption rates at the air/water interface. Furthermore, surfactants with different linking groups may display distinct dynamic behavior, even if they possess identical equilibrium surface tensions. An increase in the number of connecting carbon chains correlates with heightened flexibility of the surfactant molecules, leading to accelerated adsorption rates.

In the traditional synthesis of gemini surfactants, 1,3-propanesultone is commonly used as a sulfonating agent. However, this compound is not only difficult to obtain but also harmful to human health. To replace 1,3-propanesultone, Geng et al. [30] synthesized a series of novel gemini sulfonate betaine surfactants (DBAs-n) by reacting 1,2-bis[N-ethyl-N-(2-hydroxy-3-sulfopropyl)-alkylammonium]alkane betaine with α,ω-dibromopropane and ethyl bromide. The results indicated that the cmc of DBAs-n in aqueous solution was as low as 10^−5^ M, with the surface tension reaching a minimum of 22.2 mN/m above the cmc. These findings demonstrated that DBAs-n exhibit strong micelle-forming ability, surface activity, and encapsulation. Furthermore, it was observed that when the hydrophobic carbon chain length in DBAs-n increased from 12 to 14, the micelle morphology transitioned from vesicular to intertwined fibrous, suggesting the potential to control aggregate shapes by altering the carbon chain length. Sodium sulfanilate can serve as a connecting group in gemini surfactants, and its sulfonic acid group can also introduce anions into cationic surfactants, thereby forming zwitterionic gemini surfactants. However, the benzene ring in sodium sulfanilate makes it hydrophobic while it is part of the hydrophilic segment, complicating the prediction of its performance enhancement for single-chain cationic surfactants.

The current research on zwitterionic gemini surfactants linked by p-aminobenzenesulfonate is limited, particularly regarding their oil recovery performance and supporting data. Comprehensive evaluations of these effects are urgently needed. This study aims to synthesize the cationic surfactant N,N-dimethyl-N-(oxiran-2-ylmethyl)dodecan-1-aminium (DDPA) and the zwitterionic gemini surfactant sodium 4-[bis(3-(dodecyldimethylamino)-2-hydroxypropyl)amino]benzenesulfonate (DDBS). The molecular structures will be characterized using NMR and IR spectroscopy. The physical properties will be analyzed in terms of surface activity and thermal stability. Application performance will be assessed through interfacial activity, wettability, emulsification ability, and adsorption loss. Core flooding experiments will demonstrate the surfactants’ potential to enhance oil recovery rates. Finally, the synergistic performance of DDBS will be evaluated. This research provides insights for selecting surfactants and informs future development efforts. The experimental results and discussion are presented in Section 2, with the detailed materials and methods described in Section 3.

## 2. Results and Discussion

### 2.1. Synthesis of the Surfactant

**Figure 1:**
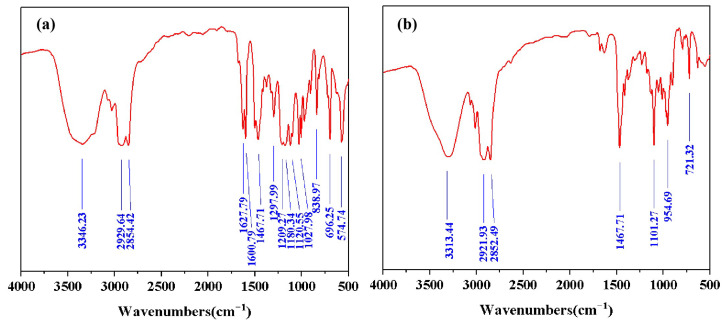
FT-IR spectra of DDPA (**a**) and DDBS (**b**).

**Figure 2:**
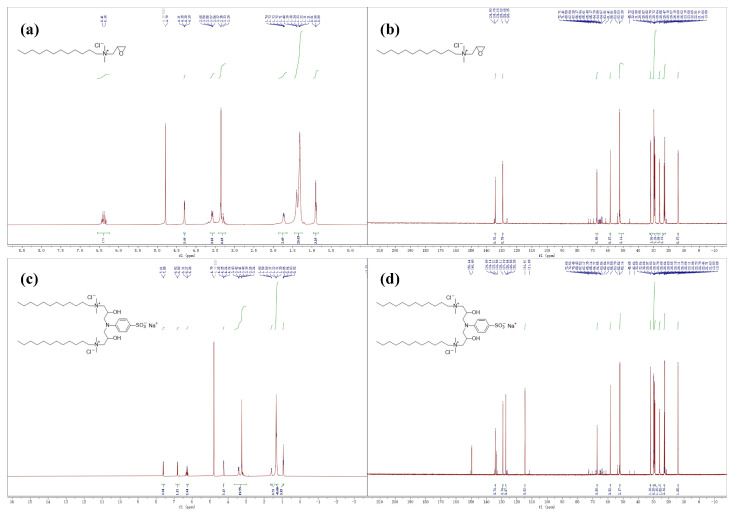
NMR spectra of DDPA and DDBS. (**a**) The ^1^H NMR spectra of DDPA. (**b**) The ^13^C NMR spectra of DDPA. (**c**) The ^1^H NMR spectra of DDBS. (**d**) The ^13^C NMR spectra of DDBS.

The structures of DDPA and DDBS were supported by Fourier Transform Infrared Spectroscopy (FT-IR) (Figure 1), ^1^H NMR and ^13^C NMR (Figure 2). As shown in Figure 1a, the absorption peak at 3313.44 cm^−1^ may correspond to the O-H stretching vibrations from water and isopropanol solvents used in the synthesis. The peaks at 2921.93 cm^−1^ and 2852.49 cm^−1^ correspond to the stretch vibration absorption associated with C-H, -CH_3_, and -CH_2_-. The peak at 1467.71 cm^−1^ corresponds to the in-plane bending vibration of the C-H bond. The peak at 1101.27 cm^−1^ corresponds to the antisymmetric stretching vibration of the epoxy bond. The absorption peak at 954.69 cm^−1^ corresponds to the symmetric stretching vibration of the epoxy bond.The peak at 721.32 cm^−1^ corresponds to the out-of-plane bending vibration of N-H.

In addition to the absorption peaks identical to those shown in Figure 1a, the infrared spectra in Figure 1b reveal that the absorption peak at 3346.23 cm^−1^ corresponds to the stretching vibration of O-H Furthermore, the additional peaks are at 1627.79 cm^−1^ and 1600.79 cm^−1^, which are associated with the C=C stretching vibrations of the phenyl ring. The absorption peaks at 1205 cm^−1^, 1180.34 cm^−1^ and 1120.55 cm^−1^ correspond to the symmetric and asymmetric stretching vibrations of -${\mathrm{SO}}_{3}^{2-}$. The peak at 1027.98 cm^−1^ corresponds to the C-O stretching vibration of the hydroxyl group. The absorption peak at 838.97 cm^−1^ corresponds to the para substitution of the benzene ring. The peak at 696.25 cm^−1^ corresponds to the out-of-plane bending vibration of the C-H bond on the benzene ring. The absorption peak at 574.74 cm^−1^ corresponds to the stretching vibration of -${\mathrm{SO}}_{3}^{-}$.

NMR testing by Bruker Avance III HD at 500 MHz was performed under the following conditions: detection temperature (295 ± 0.1) K, and the spectral widths of ^1^H-NMR and ^13^C-NMR of 8 kHz and 30 kHz, respectively. The detailed ^1^H NMR results are shown in Figure 2a,c: the proton signal at 4.7 ppm is due to the solvent protons (D_2_O). For Figure 2a, the following assignments are made: 6.38 ppm (d, 2H, **CH**_2_(O)CH-CH_2_-), 4.30 ppm (dd, 1H, CH_2_(O)**CH**-CH_2_-N-), 3.64–3.53 ppm (m, 2H, CH_2_(O)CH-**CH**_2_-N-), 3.35 ppm (s, 6H, -CH_2_-N**(CH**_2_**)**_2_-CH_2_-), 1.86–1.64 ppm (m, 2H, -N-**CH**_2_-(CH_2_)_10_-CH_3_), 1.45–1.25 ppm (m, 20H, -N-CH_2_-**(CH**_2_**)**_10_-CH_3_), and 0.90 ppm (t, 3H, N-CH_2_-(CH_2_)_10_-**CH**_3_).

For Figure 2c, the following assignments are made: 7.60 ppm (d, 2H, 2,6-Ar**H**), 6.81 ppm (d, 2H, 3,5-Ar**H**), 6.32–6.20 ppm (m, 2H, -N-CH_2_-CH**(OH)**-CH_2_-N-), 4.24 ppm (dd, 2H, -N-CH_2_-**CH**(OH)-CH_2_-N-), 3.49–3.03 ppm (m, 20H, -CH_2_-N**(CH**_3_**)**_2_-CH_2_-(CH_2_)_10_-CH_3_, -**CH**_2_-N(CH_3_)_2_-CH_2_-(CH_2_)_10_-CH_3_, -N-**CH**_2_-CH(OH)-CH_2_-N-), 1.65–1.54 ppm (m, 4H, -N-**CH**_2_-(CH_2_)_10_-CH_3_), 1.32 ppm (d, 40H, -N-CH_2_-**(CH**_2_**)**_10_-CH_3_), and 0.95–0.90 ppm (m, 6H, -N-CH_2_-(CH_2_)_10_-**CH**_3_).

The detailed ^13^C NMR results are shown in Figure 2b,d. For Figure 2b, the following assignments are made: 134.16, 129.22, 67.28, 58.51, 52.28, 32.00, 30.94–27.80, 26.05, 22.85, and 13.88. For Figure 2d, the following assignments are made: 134.14, 129.11, 127.19, 114.61, 67.17, 58.50, 52.14, 32.06, 30.43–29.37, 29.18, 25.99, 22.84, and 13.91.

The synthesis methods employed for the production of DDPA and DDBS, as validated through the combined application of FT-IR and NMR spectroscopy, are capable of producing compounds with distinctly defined molecular structures.

### 2.2. Thermal Stability

**Figure 3:**
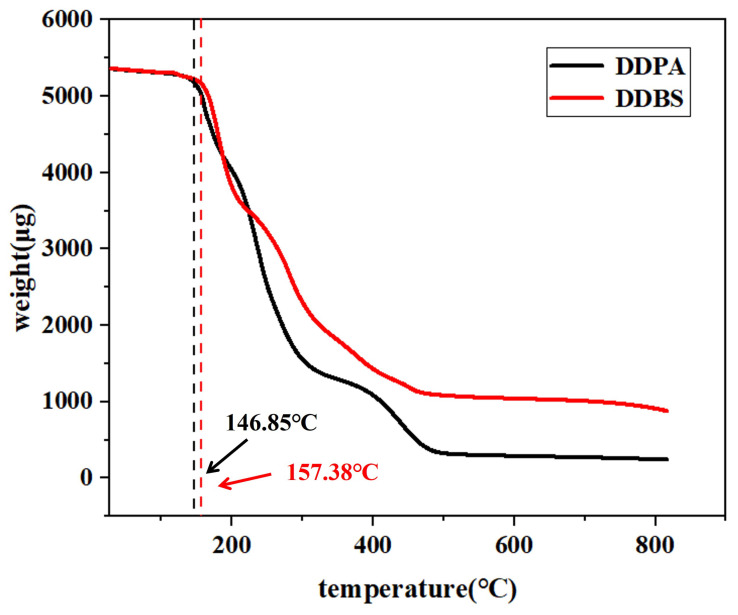
The TGA of DDPA and DDBS.

The thermogravimetric analysis (TGA) of the two surfactants is depicted in Figure 3. The figure reveals a minor mass reduction for both surfactants at low temperatures, suggesting the evaporation of isopropanol and water molecules from the samples. This could be attributed to the solvent molecules being encapsulated by surfactant molecules, hindering their removal during purification. The analysis indicates that the thermal decomposition temperature of DDPA is 146.85 °C, whereas DDBS exhibits a significantly higher temperature of 157.38 °C, reflecting the unique structural benefits of gemini surfactants. The presence of a linker enhances both the intramolecular and intermolecular interactions, conferring improved thermal stability. Given that the formation temperatures of most oil fields fall within the 50–150 °C range [31,32], both synthesized surfactants are potential candidates for oil displacement applications.

### 2.3. Surface Tension

**Figure 4:**
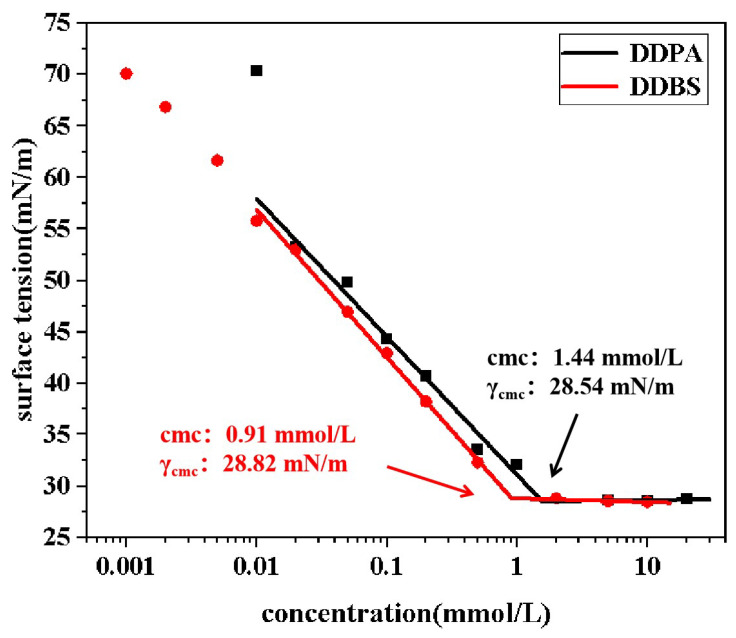
Curve of the surface tension of DDPA and DDBS with concentration.

The γ-logc curves in Figure 4 illustrate the surface tension of two surfactant solutions as a function of concentration. It is observed that at low concentrations, the surface tension of the surfactant solutions decreases with increasing molecular concentration. Upon reaching a critical concentration, the surface tension plateaus and becomes independent of further concentration changes. This critical concentration is known as the cmc, and the corresponding surface tension is the minimum surface tension ($\gamma_{cmc}$) achievable by the surfactant.

From the data presented, the cmc values for DDBS and DDPA are 0.91 mmol/L and 1.44 mmol/L, respectively, with corresponding $\gamma_{cmc}$ values of 28.82 mN/m and 28.54 mN/m. The connecting group incorporated in DDBS may enhance the surfactant’s surface adsorption and aggregation tendencies at low concentrations, thereby rendering the influence of the concentration on the surface tension more pronounced. However, this observation is based on a limited dataset and thus requires cautionary interpretation. Additionally, it lowers the cmc, allowing the surfactant to reach optimal conditions at lower concentrations. However, it does not reduce the $\gamma_{cmc}$ to a lower level, suggesting that DDBS and DDPA share a similar mechanism in reducing the surface tension.

Calculations show that both DDPA and DDBS have negative standard micellization free energy ($\Delta G_{mic}^{0}$) and standard adsorption free energy ($\Delta G_{ads}^{0}$). This indicates that the adsorption of these surfactants at the gas/liquid interface and their micelle formation in the bulk solution are spontaneous processes [33]. Additionally, the $\Delta G_{ads}^{0}$ is lower than the $\Delta G_{mic}^{0}$ for both surfactants, suggesting that they preferentially adsorb at the interface. Once saturation is reached, they form aggregates in the solution. See Supplementary Materials Table S1 for the specific calculation formula and results.

### 2.4. Interfacial Tension

#### 2.4.1. Effect of Concentration on Interfacial Tension

**Figure 5:**
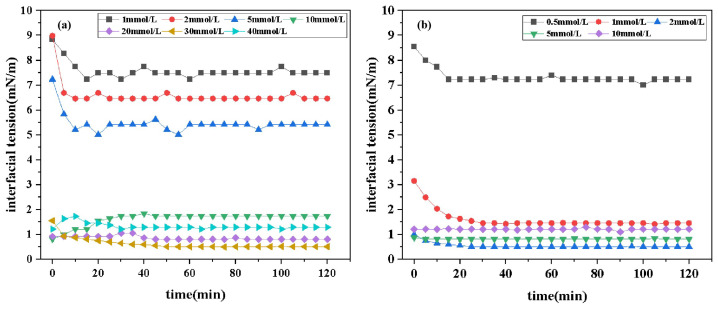
Interfacial tension curves of DDPA (**a**) and DDBS (**b**) at various concentrations.

Figure 5 presents the interfacial tension (IFT) curves of two surfactants, DDPA and DDBS, at varying concentrations over time. The data reveal that both surfactants effectively reduce the oil–water IFT, with minimum interfacial tensions (IFT_*min*_) of 0.5128 mN/m and 0.5122 mN/m, respectively. This indicates good compatibility with Xinjiang crude oil. Comparative analysis shows that the cationic surfactant DDPA requires a concentration of 3 × 10^−2^ mol/L to achieve IFT_*min*_, while the gemini surfactant DDBS can achieve the same effect at a lower concentration of 2 × 10^−3^ mol/L, demonstrating significantly enhanced interfacial activity and reduced economic costs.

Furthermore, the relationship between the IFT and concentration over time shows an “L” shaped trend, where the oil–water IFT rapidly decreases to a minimum value and then gradually stabilizes. Thus, for both DDPA and DDBS, the IFT_*min*_ equals the equilibrium interfacial tension (IFT_*equ*_). This phenomenon occurs because, initially, the concentration of surfactant molecules in the bulk phase is much lower than that at the interface. Surfactant molecules preferentially diffuse from the solution interior to the interface, where the rate of interfacial adsorption exceeds the rate of desorption, leading to a gradual reduction in system IFT. When adsorption and desorption reach equilibrium, the IFT stabilizes. The observed phenomena align with those reported in the literature [34]. This effect is particularly evident at lower surfactant concentrations. As the concentration of surfactants increases, the adsorption rate of molecules at the interface significantly accelerates, leading to rapid saturation. Concurrently, micelles form at the interface during adsorption, which obscures the “L”-shaped trend, making it less discernible.

Additionally, comparing the IFT_*min*_ values of DDPA and DDBS reveals that they are very close, suggesting a commonality in their mechanisms for reducing IFT, consistent with previous experimental Section 2.3 results on surface tension.

#### 2.4.2. Effect of Temperature on Interfacial Tension

**Figure 6:**
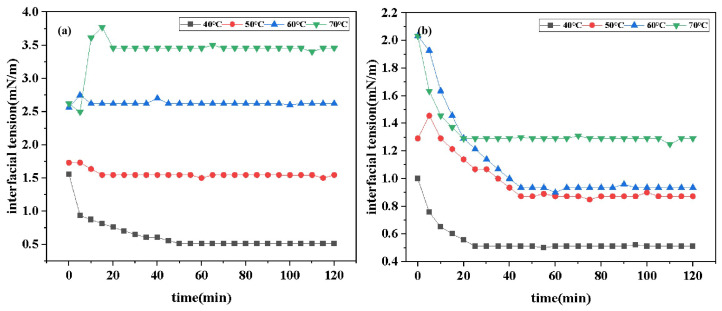
Interfacial tension curves of DDPA (30 mmol/L) (**a**) and DDBS (2 mmol/L) (**b**) at various temperatures.

Figure 6 shows the time-dependent curves of IFT of two surfactants at different temperatures. Data derived from the graph indicate that the IFT_*min*_ of DDPA at 40 °C, 50 °C, 60 °C, and 70 °C are 0.5128 mN/m, 1.5454 mN/m, 2.6194 mN/m, and 3.4558 mN/m, respectively. Similarly, the IFT_*min*_ of DDBS at these temperatures are 0.5122 mN/m, 0.8729 mN/m, 0.9352 mN/m, and 1.2917 mN/m. Comparative analysis reveals that the IFT of both surfactants increases with rising temperature. This trend is attributed to two primary factors: firstly, the increase in temperature alters the partition coefficient of surfactants in the oil phase, leading to a transition from a dense to a more sparse molecular arrangement at the interface; secondly, the adsorption and desorption rates of the interfacial molecules increase with temperature, which compromises the stability of the interfacial film. Additionally, the long carbon chains of surfactant molecules may deform and aggregate at high temperatures, resulting in decreased hydrophobicity and reduced contact area with the oil phase, thereby increasing the IFT [35]. These findings are consistent with the observations reported in the literature, confirming the influence of temperature on the surfactant behavior and interfacial tension. Furthermore, the data comparison shows that the increase in the IFT_*min*_ of DDBS with temperature is significantly lower than that of DDPA. This is attributed to the more stable interfacial film structure being formed by gemini surfactants and the incorporation of the sulfonate group from aminobenzene sulfonic acid into DDBS, which imparts excellent thermal stability to the anionic surfactant, aligning with the research expectations.

#### 2.4.3. Effect of Inorganic Salt Concentration on Interfacial Tension

**Figure 7:**
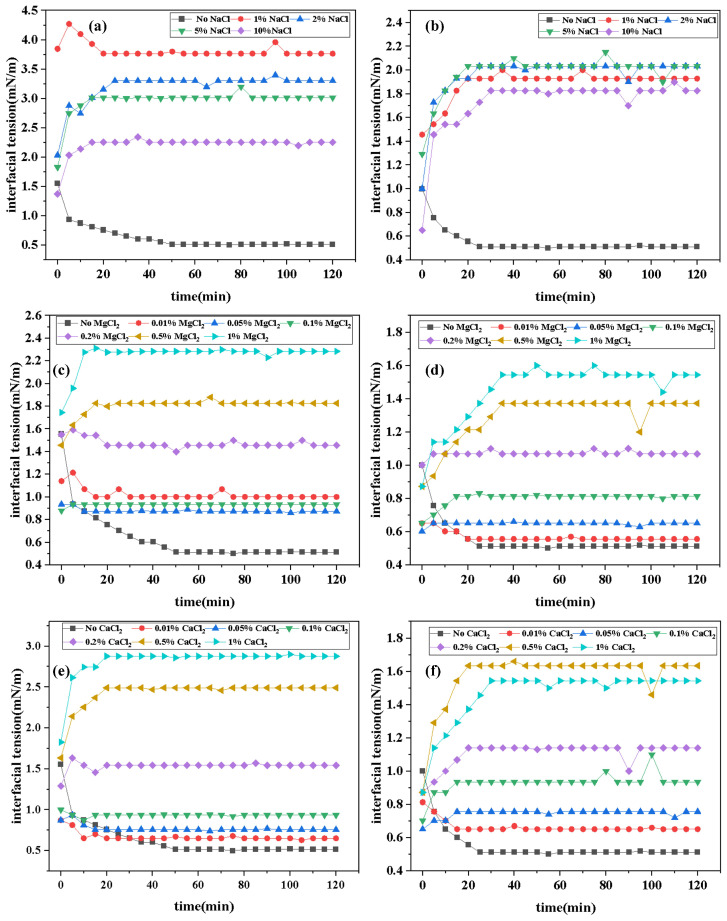
Interfacial tension curves of DDPA (30 mmol/L) and DDBS (2 mmol/L) in different inorganic salt environments. (**a**) DDPA in in sodium chloride environment. (**b**) DDBS in sodium chloride environment. (**c**) DDPA in magnesium chloride environment. (**d**) DDBS in magnesium chloride environment. (**e**) DDPA in calcium chloride environment. (**f**) DDBS in calcium chloride environment.

Figure 7 presents the curves of IFT variation over time for two surfactants under different concentrations of inorganic salts. Data from the figure indicate that the addition of inorganic salts increases the equilibrium IFT_*equ*_ values of surfactants. However, the experimental results do not exhibit a simple trend of “IFT with increasing cation concentration”. Instead, the salt concentration–IFT curves transition from an “L” shape to a “Γ” shape, indicating that IFT gradually rises to a plateau value and then stabilizes (this phenomenon is more pronounced at higher salt concentrations). This suggests that the interaction between cations and surfactants involves complex mechanisms, possibly including synergistic effects within certain concentration ranges. The reasons for this phenomenon may include the following: in the absence of added salts, surfactants are loosely arranged at the oil–water interface. When a certain amount of salt is added, inorganic salt ions aggregate near the hydrophilic head groups of surfactants, reducing the electrostatic repulsion between hydrophilic groups and compressing the surfactant double layer. This results in a more compact aggregation structure of surfactants, increasing the close packing of surfactant molecules per unit area at the oil–water interface, thereby reducing the IFT. When a large amount of salt is added, the high salt concentration shields ion charges, disrupts the hydration layer around ions, increases the hydrophobicity of surfactants, facilitates micelle formation, and induces salting-out effects, leading to more surfactant molecules dissolving in the oil phase. This reduces the effective concentration of surfactants at the interface, disrupting the adsorption equilibrium between the water and oil interfaces and increasing IFT. The observed phenomena align with those reported in the literature [36,37,38]. Comparative data analysis reveals that the increase in IFT of DDBS with an inorganic salt concentration is significantly lower than that of DDPA. This is not only due to the more stable interfacial film formed by gemini surfactants, but also, more importantly, the introduction of anionic hydrophilic groups forms a structure similar to an internal salt, reducing the interference of environmental electrolytes on surfactants. Therefore, DDBS exhibits better inorganic salt tolerance, which aligns with the experimental expectations.

### 2.5. Wettability

**Figure 8:**
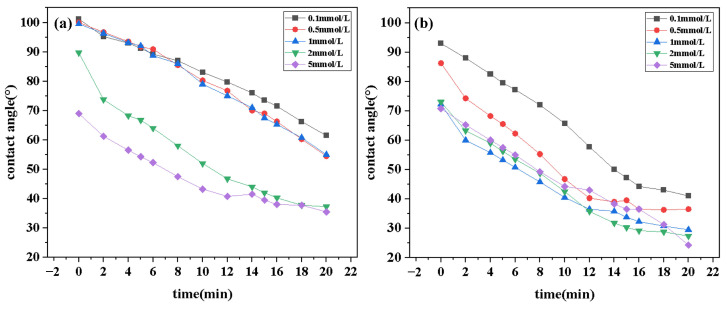
Contact angle curves of DDPA (**a**) and DDBS (**b**).

Figure 8 illustrates the contact angle–time curves for aqueous solutions of DDPA and DDBS at different concentrations at 25 °C. Data from the figure reveals that DDPA and DDBS can reduce the contact angles to 35.5° and 24.5°, respectively, within 20 min, indicating a pronounced wetting reversal phenomenon. Both samples exhibit strong wetting properties, with DDBS outperforming DDPA in both the rate and extent of the contact angle reduction, especially at low concentrations, which is in line with the experimental expectations. Observations of the curve characteristics show that in the initial stage of sample droplet contact with the paraffin film, the contact angle decreases rapidly; DDBS can reduce the contact angle to below 80° within seconds, completing the wetting reversal process. Over time, the rate of the contact angle reduction gradually stabilizes as the adsorption and desorption at the solid–liquid interface reach a dynamic equilibrium. Furthermore, as the concentration of surfactants increases, the overall contact angle of the droplet decreases. When the concentration exceeds the cmc of the samples, the variation between curves at different concentrations becomes significantly smaller, indicating that the surfactants have entered their optimal working state.

### 2.6. Emulsibility

Table 1 illustrates the variation in emulsion duration times for DDPA and DDBS solutions with varying concentration and temperature. Analysis of the data reveals that at a concentration of 0.25 g/L, DDPA exhibits the longest emulsion duration time, reaching up to 503 s, while DDBS shows the longest emulsion duration time of 262 s at a concentration of 0.30 g/L. As the concentration of the surfactants increases, the emulsion duration times initially rise and then decrease. This phenomenon may be attributed to the fact that at low surfactant concentrations, fewer molecules adsorb at the oil–water interface, resulting in a loose and less stable interfacial film. With increasing surfactant concentration, more molecules accumulate at the interface, forming a denser and stronger film, which enhances the stability of the emulsion system. However, further increases in the surfactant molecules lead to aggregation and association within the bulk phase, causing the oil droplets dispersed in the aqueous phase to flocculate and settle, reducing the time required for demulsification. Comparative data across different temperatures show that as the temperature gradually increases, the emulsion duration times also decrease. This is due to the reduced system viscosity and intensified Brownian motion at higher temperatures, increasing the likelihood of oil droplet contact and thus decreasing the overall stability of the emulsion. Comparing the data for DDPA and DDBS, DDPA demonstrates significantly better emulsion stability than DDBS. This is speculated to be due to the larger volume of DDBS as a gemini surfactant, which increases the probability of contact between oil droplets, and the presence of the benzene hydrophobic group in sodium sulfanilate, which weakens the hydrophilicity of the hydrophilic group, affecting the water solubility of DDBS molecules and reducing the overall stability of the emulsion. The observed trends in emulsion stability align with the mechanisms described in the literature [39], confirming the validity of the proposed explanations. However, in actual oil displacement processes, the emulsion duration times need to be coordinated with the time of the surfactant displacement agents in the reservoir to avoid difficulties in demulsification. Therefore, the practical value of DDBS and DDPA must be determined based on specific usage scenarios.

### 2.7. Adsorptivity

Quartz sand surfaces are characterized as high-energy hydrophilic surfaces with a surface free energy of approximately 76 mJ/m^2^. The adsorption amount of the surfactant onto the Quartz sand can be calculated based on the difference between the initial concentration (*C*_0_) and the equilibrium concentration (*C*) of the surfactant before and after measurement, using the following formula [40]:

(1) $$\Gamma =(\left(C_{0}-C\right)V\times 10^{-3})/M$$

where *C*_0_ is the initial concentration in mg/L, *C* is the equilibrium concentration in mg/L, *V* is the solution volume in mL, and *M* is the mass of quartz sand in g.

**Figure 9:**
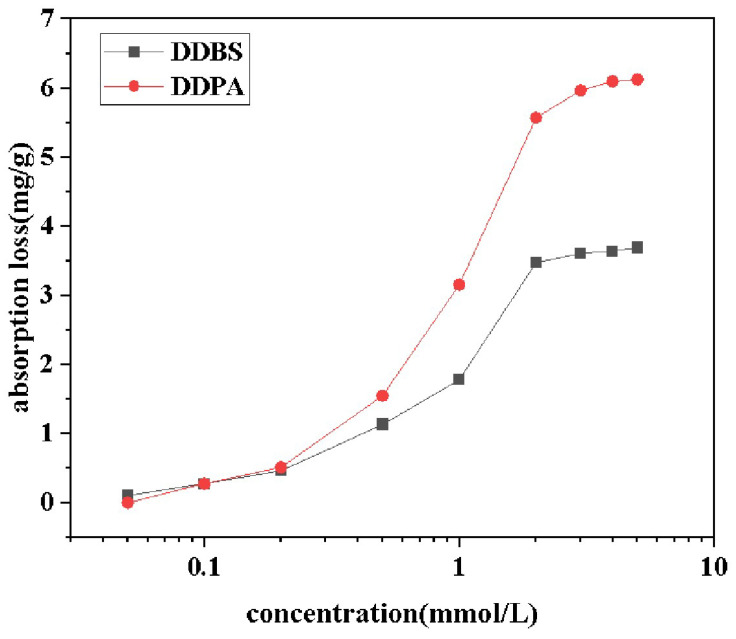
Absorption loss curves of DDPA and DDBS.

The adsorption of surfactants onto solid surfaces is primarily influenced by interactions such as electrostatic forces, van der Waals forces, hydrogen bonds, and hydrophobic interactions [41,42]. Due to the presence of both positive and negative charge centers in DDBS, its interaction with solid surfaces is more complex compared to conventional ionic surfactants, and cannot be explained by a single adsorption mechanism [43]. Data from Figure 9 indicate that the adsorption curves of both surfactants generally conform to the S-shaped isotherm adsorption model, suggesting that the adsorption on the negatively charged interface is not a simple monolayer adsorption but rather the formation of a new, looser adsorption layer through hydrophobic interactions. At low surfactant concentrations, surfactant molecules primarily adsorb onto the negatively charged surface through electrostatic interactions, with these monomers serving as nucleation points for the further adsorption of surfactant molecules, designating this region as the electrostatic zone. As the surfactant concentration increases, active sites on the quartz sand are gradually occupied by the molecules until adsorption saturation is reached. Concurrently, the hydrophobic groups of the surfactants and newly induced charge sites on the surface become nucleation points for further adsorption, with this stage being driven by both electrostatic and hydrophobic interactions, thus classifying this region as the electrostatic–hydrophobic zone. When the surfactant concentration further increases, any additional adsorption is driven purely by hydrophobic interactions, while resisting repulsive electrostatic barriers due to overcompensation of the surface charge by the surfactant. Additionally, due to steric hindrance and the formation of micelles in the solution by excess molecules, surfactant molecules reach an adsorption–desorption equilibrium at the solid–liquid interface, with the adsorption amount remaining almost unchanged, designating this region as the hydrophobic zone. Comparative analysis of the data reveals that the maximum adsorption capacities of DDPA and DDBS are 6.12 mg/g and 3.69 mg/g, respectively, with DDBS exhibiting a significantly lower adsorption loss, thereby holding a stronger competitive edge in enhanced oil recovery. The reasons for this phenomenon include the spatial steric hindrance due to the large molecular volume of DDBS and the introduction of negative charges in the connecting group, which generates electrostatic repulsion with the quartz sand surface.

### 2.8. Oil Displacement Capability

**Figure 10:**
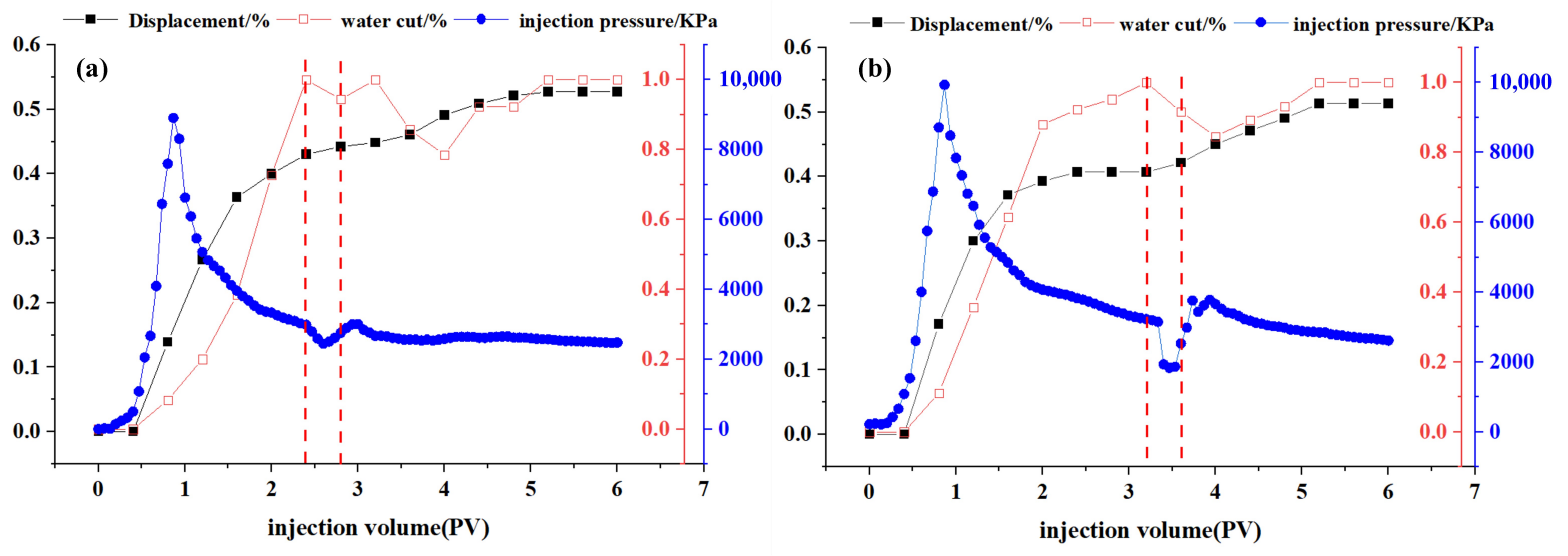
Curves of displacement, water cut and injection pressure versus injection volume for DDPA (**a**) and DDBS (**b**) at 40 °C.

The recovery efficiency, water cut, and injection pressure of DDPA and DDBS surfactants as a function of injection volume are depicted in Figure 10, with the vertical red dashed lines indicating the corresponding volumes at which the surfactant solutions begin and cease injection. Analysis of the curve characteristics reveals a distinct inflection in the injection pressure curve following the surfactant drive, attributed to the surfactant’s actions such as emulsification, wettability alteration, and reduction in IFT, which mobilize the residual oil from pore channels, fissures, or rock surfaces, forming larger oil bands. These bands increase the displacement pressure during their migration, with the pressure gradually stabilizing once the oil bands are displaced. Data analysis indicates that, compared with water, DDPA can enhance oil recovery efficiency by 8.5%, while DDBS can increase it by 7.9%. Although the oil recovery enhancement is similar for both surfactants, DDBS achieves equivalent displacement at a significantly lower concentration of 2 mmol/L, compared to the 30 mmol/L of DDPA, thereby substantially reducing costs. Therefore, it can be inferred that DDBS possesses superior oil displacement capabilities, effectively improving the recovery efficiency and holding considerable potential for application in oilfield development under conditions of high temperature and high mineralization.

### 2.9. Compatibility

**Figure 11:**
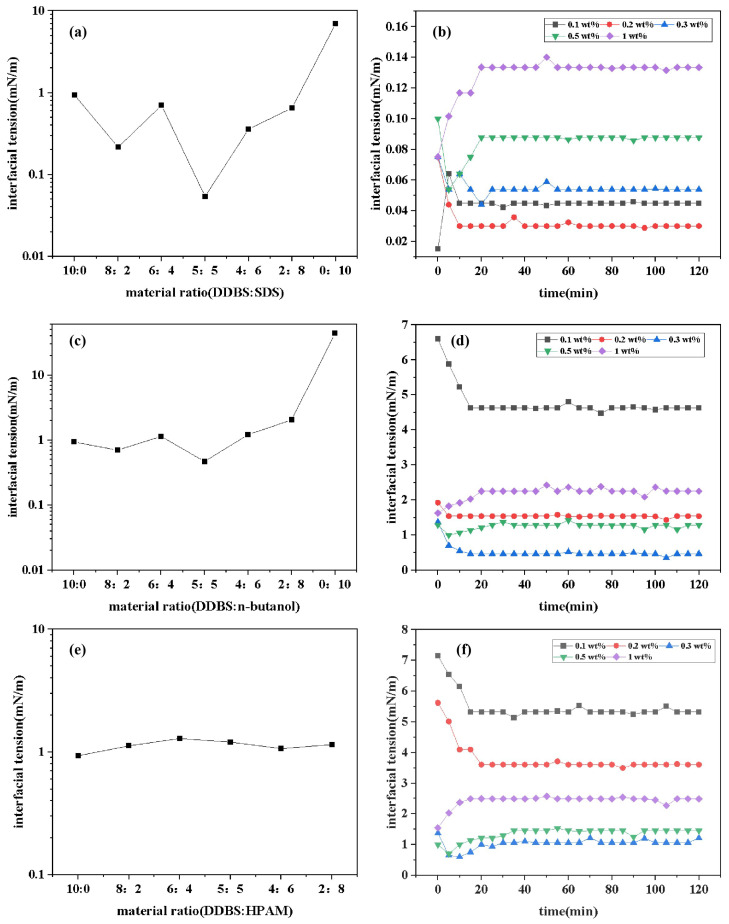
Interfacial tension curves of different compound systems. (**a**) Interfacial tension of DDBS/SDS under different material ratios. (**b**) Time-dependent curve of the interfacial tension of DDBS/SDS. (**c**) Interfacial tension of DDBS/n-butanol under different material ratios. (**d**) Time-dependent curve of interfacial tension of DDBS/n-butanol. (**e**) Interfacial tension of DDBS/PAM under different material ratios. (**f**) Time-dependent curve of interfacial tension of DDBS/PAM.

The interfacial tension values of the DDBS/SDS, DDBS/n-butanol, and DDBS/ polyacrylamide (PAM) mixed systems with different ratios are depicted in Figure 11a,c,e. Data analysis reveals that the IFT_*min*_ are achieved at ratios of DDBS:SDS = 5:5, DDBS:n-butanol = 5:5, and DDBS:PAM = 4:6, with values of 0.05539 mN/m, 0.4700 mN/m, and 1.068 mN/m, respectively. These ratios are identified as the optimal formulations for their respective mixed systems. The dynamic IFT of the DDBS/SDS, DDBS/n-butanol, and DDBS/PAM mixed systems at various concentrations over time is illustrated in Figure 11b,d,f. Data analysis reveals that for the DDBS/SDS mixed system, at a mass ratio of 5:5 and a concentration of 0.2 wt%, the IFT can be reduced to a minimum of 0.0301 mN/m. For the DDBS/n-butanol mixed system, at a mass ratio of 5:5 and a concentration of 0.3 wt%, the IFT can be minimized to 0.4700 mN/m. For the DDBS/PAM mixed system, at a mass ratio of 4:6 and a concentration of 0.3 wt%, the IFT can be lowered to 1.068 mN/m. Notably, the DDBS/SDS system can reduce the IFT_*min*_ value of the single-component DDBS by an order of magnitude, significantly enhancing the performance of the system and demonstrating substantial potential for application. This phenomenon is attributed to the fact that DDBS, although a zwitterionic surfactant, contains discrete charges within its structure, with a predominance of positive charges over negative charges. This characteristic results in DDBS having a strong water solubility and a propensity for strong synergistic interactions with ionic surfactants. SDS, as an anionic sulfonate surfactant, exhibits much stronger synergistic effects compared to other types of hydrophilic head groups of cationic and nonionic surfactants [44]. A substantial body of literature has demonstrated that when the oil–water IFT is reduced to the order of 10^−2^ mN/m, surfactant flooding agents can effectively enhance the recovery of crude oil. This evidence underscores the potential of the DDBS/SDS mixed system to further augment the application of DDBS in actual crude oil extraction.

## 3. Materials and Methods

### 3.1. Materials

The N,N-dimethyldodecylamine used in this study was sourced from Shanghai Titan Technology Co., Ltd. (Shanghai, China), with a purity of 97%; analytical-grade epichlorohydrin, ethyl acetate, and n-butanol were obtained from Shanghai Boer Chemical Reagent Co., Ltd. (Shanghai, China); 4-amino-benzenesulfonic acid monosodium salt, with a purity of 97%, was procured from Shanghai Bind Medicinal Technology Co., Ltd. (Shanghai, China); analytical-grade isopropanol was supplied by Beijing Yili Fine Chemicals Co., Ltd. (Beijing, China); analytical-grade sodium chloride (NaCl) was provided by Fuchen (Tianjin) Chemical Reagent Co., Ltd. (Tianjin, China); 96% calcium chloride (CaCl_2_) and 98% magnesium chloride (MgCl_2_) were acquired from Tianjin Guangfu Science and Technology Development Co., Ltd. (Tianjin, China); analytical-grade potassium chloride (KCl) of guaranteed purity was obtained from Shanghai Aladdin Biochemical Technology Co., Ltd. (Shanghai, China); analytical-grade sodium dodecyl sulfate was sourced from Beijing InoKai Technology Co., Ltd. (Beijing, China); analytical-grade polyacrylamide (PAM) was purchased from Chengdu Aikod Chemical Reagent Co., Ltd. (Chengdu, China); analytical-grade quartz sand (80–120 mesh) was supplied by Beijing Jianqiang Weiye Technology Co., Ltd. (Beijing, China); paraffin film was supplied by Bemis Company, Inc.; and dehydrated and degassed crude oil was obtained from Xinjiang Oilfield, with specific parameters detailed in Supplementary Materials Table S2.

#### 3.1.1. Synthesis of the Surfactant DDPA

**Figure 12:**
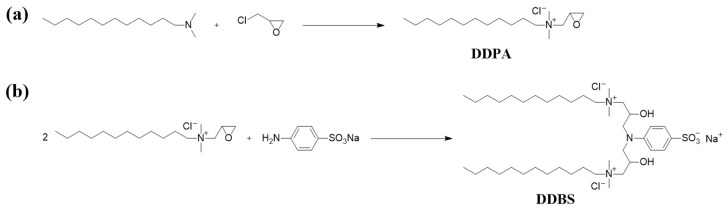
Synthesis of the surfactants DDPA (**a**) and DDBS (**b**).

To synthesize DDPA, 0.05 mol (10.67 g) of dodecyl dimethyl tertiary amine was dissolved in 75 mL of isopropanol and the solution was transferred to a 250 mL three-necked flask. Under heating and magnetic stirring, epichlorohydrin (0.055 mol, 5.09 g) was added dropwise to the flask in 0.5 h, maintaining a temperature of 80 °C for a period of 8 h. Once the reaction was complete, the reaction mixture was transferred to a round-bottom flask and rotary evaporation was performed under reduced pressure (65 °C, 0.09 MPa) to remove the excess epichlorohydrin and isopropanol. Subsequently, the crude product was placed in a vacuum drying oven and dried at 60 °C for 72 h to yield a light yellow, semi-transparent viscous solid of DDPA with a yield of up to 90% (Figure 12a).

#### 3.1.2. Synthesis of the Surfactant DDBS

To synthesize DDBS, commenced by dissolved DDPA (0.025 mol, 7.648 g) and 4-amino-benzenesulfonic acid monosodium salt (0.0125 mol, 2.44 g) in 50 mL of deionized water within a 250 mL three-necked flask. Concurrently, we introduced 30 mL of isopropanol into a separate flask and heated the mixture to 90 °C, stirring for 6 h to ensure complete reaction. Post-reaction, we transferred the mixture to a round-bottom flask and employed rotary evaporation under reduced pressure (35 °C, 0.09 MPa) to evaporate the isopropanol. The crude product, following rotary evaporation, was then washed thrice with ethyl acetate to remove impurities, and then subjected to vacuum rotary evaporation (40 °C, 0.09 MPa) to eliminate the residual ethyl acetate and water. Finally, the crude product was placed in a vacuum drying oven and dried at 60 °C for 72 h to yield a yellow–brown viscous solid of DDBS yielding up to 90% (Figure 12b).

### 3.2. Characterization

The structure of the product was characterized by FT-IR,^1^H-NMR and ^13^C-NMR. The FT-IR measurements were conducted using a MAGNA-IR560E.S.P spectrometer (Aurora, ON, Canada). The experimental conditions were as follows: a resolution of 4 cm^−1^ and a sample scanning time of 32 s. Data acquisition was performed over the spectral range from 4000 cm^−1^ to 400 cm^−1^. NMR testing was performed by Bruker (Billerica, MA, USA) Avance III HD at 500 MHz under the following conditions: detection temperature (295 ± 0.1) K, and spectral widths of ^1^H-NMR and ^13^C-NMR of 8 kHz and 30 kHz, respectively.

### 3.3. Thermal Stability

The thermal stability of the surfactants was tested by a HTG-3 thermogravimetric analyzer. The alumina crucible used in the experiment was calcined at 800 °C for 2 h in a muffle furnace, and 5.4 mg of surfactant sample was weighed and placed in the alumina crucible, while an empty crucible was placed as a control. At the beginning of the experiment, we opened the condensate valve and introduced argon gas into the sample at a rate of 50 mL/min. We slowly raised the temperature to 800 °C with a heating rate of 10 °C/min. We recorded the changes in the sample mass during the heating process.

### 3.4. Surface Tension

Surface tension measurements of the surfactants were conducted using a BZY-2 surface tension (Shanghai Weichuan Precision Instrument Co., Ltd., Shanghai, China) meter employing the Wilhelmy plate technique. The watch glass containing the sample solution was positioned on the lifting platform, with the Wilhelmy plate suspended directly above it. Upon activation of the platform, the solution within the watch glass made contact with the plate, triggering an automatic cessation of the platform’s ascent. Once the instrument reading had stabilized, the surface tension value γ was recorded. A series of surfactant solutions with varying concentrations were measured, and the average of three replicate measurements at 25 °C was taken for each concentration. Following each measurement, the platinum plate was removed, rinsed with deionized water, heated to red heat using an alcohol spray gun for over ten seconds to eliminate any residual substances, and then allowed to cool to room temperature before the next set of samples was measured.

### 3.5. Interfacial Tension

The interfacial tension of surfactants was determined using a TX-500C (Shanghai Zhongchen Digital Technology Co., Ltd., Shanghai, China) rotary droplet interfacial tension meter, which employs the spin drop method. This method calculates interfacial tension based on the shape and size of the droplet (or bubble), utilizing the Bashforth–Adams equation. Before the experiment, a series of surfactant solutions with varying concentrations were prepared and sonicated for 30 min. The treated sample solution was precisely transferred into a glass quartz tube. Subsequently, a measured quantity of Xinjiang crude oil was introduced into the tube, enabling it to be suspended within the solution. For the testing procedure, the TX-500C instrument was preheated to the specified testing temperature, with a rotation speed of 6000 rpm. Images were captured at 5 min intervals over a testing period of 2 h. By varying the temperature and the type and concentration of cations, the temperature and salt resistance properties of the surfactants were systematically investigated.

### 3.6. Wettability

The wettability of the surfactants was quantified via contact angle measurements using the JC2000 instrument (Shanghai Zhongchen Digital Technology Co., Ltd., Shanghai, China). Paraffin film, simulating hydrophobic rock surfaces, was used as the substrate. A 5 μL droplet of surfactant solution was applied, and photographs were taken every 2 min for 20 min to track the droplet behavior. Triplicate measurements were averaged for each concentration. To minimize evaporation, a square glass cover was placed over the droplets. Pure water served as a control, showing negligible changes in volume and contact angle over time. The syringe was cleaned between sample changes. See Supplementary Materials Table S3 for the water control experiment data.

### 3.7. Emulsibility

The emulsifying efficacy of the surfactants was evaluated following the emulsification and water separation protocol outlined in reference [45]. Surfactant solutions of varying concentrations were mixed with crude oil in a 10 mL graduated cylinder at a 6:4 volume ratio and equilibrated in a 25 °C water bath. The cylinder was then vigorously inverted five times, allowed to stand for 1 min, and this process was repeated five times in total. Post-shaking, the cylinder was placed in a water bath at the specified test temperature, and a stopwatch was used to measure the time required for 1 mL of water to separate from the emulsion. This procedure was conducted thrice, with the average time recorded.

### 3.8. Adsorptivity

The adsorptive capacity of surfactants was evaluated through static adsorption experiments, using quartz sand to simulate reservoir conditions. Surfactant solutions of varying concentrations were prepared. A Mettler Toledo s230 meter recorded conductivity for a calibration curve [46]. In 50 mL vials, 1 g of quartz sand was mixed with 10 mL of surfactant solutions and shaken in a 30 °C water bath at 150 rpm for 24 h. The samples were then centrifuged at 12,000 rpm for 25 min, and the supernatant was analyzed for conductivity. Surfactant concentrations were determined from the standard curve and used to calculate adsorption loss. See Supplementary Materials Figures S1 and S2 for conductivity–concentration calibration curves.

### 3.9. Oil Displacement Capability

The oil displacement capability of surfactants was measured with a CFS-100 displacement (Beijing Bilai Petroleum Instruments Co., Ltd., Beijing, China)apparatus at 40 °C and 8 MPa using artificial sandstone cores [47]. See Supplementary Materials Tables S4 and S5 and Section S2 for additional information regarding the artificial sandstone cores, simulated formation water, and detailed experimental procedures. The surfactant concentration was optimized as detailed in Section 3.5. The potential for oil displacement was assessed by simulating crude oil recovery increases in the experiments.

### 3.10. Compatibility

DDBS/SDS, DDBS/n-butanol, and DDBS/PAM composite systems were prepared with varying mass ratios and concentrations. Samples were sonicated for 30 min, followed by the measurement of IFT as described in Section 3.5. The composite systems’ performance was inferred from the IFT results.

## 4. Conclusions

Using sodium p-aminobenzenesulfonate as a connecting group, the synthesis of cationic monomeric surfactant DDPA and amphoteric gemini surfactant DDBS was achieved. Various tests confirmed the structure of the products. Experimental results demonstrate that DDBS exhibits superior thermal stability, interfacial activity, temperature and salt resistance, wettability, adsorption, and enhanced oil recovery capabilities compared to DDPA. This is attributed to the unique structural advantages of gemini surfactants and the anionic groups introduced by sodium sulfonate. It indicates that further synthesis steps of DDPA using sodium sulfonate as a connecting group have practical application value. Notably, DDBS can achieve the same efficacy at a concentration one-fifteenth of that of DDPA in terms of interfacial activity and oil recovery. However, both DDBS and DDPA achieve nearly identical IFT_*min*_, suggesting a certain uniformity in their mechanisms for reducing interfacial tension, which requires further investigation. In emulsification tests, DDPA shows superior emulsion stability compared to DDBS, highlighting the complexity of the impact of the contradictory hydrophobic group structure of DDBS on its performance. By forming a mixed system with DDBS and SDS, the performance of the single-component DDBS can be significantly enhanced, further increasing its potential for application in actual extraction work.

While this paper provides insights into the properties of DDPA and DDBS, there is limited investigation into the mechanisms underlying their behavior, particularly regarding the aggregation and self-assembly of surfactants. The research is currently at the laboratory stage, and it is hoped that future studies will further explore this area. In terms of molecular structure design, researchers might consider replacing sodium sodium sulfonate with structures that lack hydrophobic groups or introduce additional anionic groups onto the benzene ring to achieve charge balance in individual surfactant molecules, thereby potentially enhancing the performance ceiling of this series of structures.

## Figures and Tables

**Table 1:** DDPA and DDBS emulsification water separation table.

Sample	Temperature (°C)	0.05 g/L	0.10 g/L	0.15 g/ L	0.20 g/L
DDPA	25	321	398	485	492
DDPA	45	189	225	234	241
DDPA	75	91	106	117	128
**Sample**	**Temperature (°C)**	**0.25 g/L**	**0.30 g/L**	**0.35 g/L**	**0.40 g/L**
DDPA	25	503	450	377	-
DDPA	45	247	232	220	-
DDPA	75	141	108	98	-
**Sample**	**Temperature (°C)**	**0.05 g/L**	**0.10 g/L**	**0.15 g/L**	**0.20 g/L**
DDBS	25	209	218	232	237
DDBS	45	128	137	158	165
DDBS	75	74	78	81	84
**Sample**	**Temperature (°C)**	**0.25 g L^−1^**	**0.30 g L^−1^**	**0.35 g L^−1^**	**0.40 g L^−1^**
DDBS	25	244	255	262	256
DDBS	45	170	179	186	180
DDBS	75	87	86	90	77

## Data Availability

Dataset available on request from the authors.

## Supplementary Materials

The following supporting information can be downloaded at: https://www.mdpi.com/article/10.3390/molecules30071537/s1, Section S1. Thermodynamic properties of surfactants. Table S1 Surface activity parameters of surfactants. Table S2. Compositions and property of Xinjiang crude oil. Table S3. The contact angle data of water. Figure S1. The conductivity-concentration calibration curve of DDPA. Figure S2. The conductivity-concentration calibration curve of DDBS. Table S4. Basic parameters of sandstone cores. Section S2. Specific steps of core displacement experiment. Table S5. Composition of simulated formation water.
