# Supplementary material for: Exploring the Performance Advantages of p-Aminobenzenesulfonate-Based Zwitterionic Gemini Surfactants in Oil Recovery
Source: Molecules. 2025 Mar 30;30(7):1537. doi: 10.3390/molecules30071537 (PMC11990317; doi:10.3390/molecules30071537)
Supplement: Supplementary file 1 [file molecules-30-01537-s001.zip › molecules-3518921-supplementary.pdf]

Supporting Information

Exploring the Performance Advantages of p-Aminobenzenesulfonate-Based Zwitterionic Gemini Surfactants in Oil Recovery

Content

Section S1. Thermodynamic properties of surfactants.

Table S1 Surface activity parameters of surfactants.

Table S2. Compositions and property of Xinjiang crude oil.

Table S3. The contact angle data of water.

Figure S1. The conductivity-concentration calibration curve of DDPA.

Figure S2. The conductivity-concentration calibration curve of DDBS.

Table S4. Basic parameters of sandstone cores.

Section S2. Specific steps of core displacement experiment.

Table S5. Composition of simulated formation water.

Section S1. Thermodynamic properties of surfactants.

To investigate the thermodynamic properties of surfactants, the standard micellization free energy (ΔG_{mic}^{0}) and the standard adsorption free energy (ΔG_{ads}^{0}) can be derived from the phase separation model using the following equations:

$$\Delta G_{mic}^{0} = RTlnX_{cmc} \quad \text{(S1)}$$

$$\Delta G_{ads}^{0} = \Delta G_{mic}^{0} - (\gamma_0 - \gamma_{cmc})/\Gamma_{cmc} \quad \text{(S2)}$$

Here, X_{cmc} represents the cmc in mole fraction, calculated as X_{cmc}=cmc/55.4; γ0 denotes the surface tension of pure water; Γ_{cmc} represents the interfacial adsorption capacity of surfactants, is a crucial parameter for characterizing their adsorption state at the interface.

The adsorption of surfactants at the interface can be calculated using the Gibbs isotherm equation:

$$\Gamma = -1/(2.303nRT)(d\gamma/logC) \quad \text{(S3)}$$

Here, γ represents the surface tension; R is the gas constant with a value of 8.314 $J \cdot mol^{-1} \cdot K^{-1}$; T is the absolute temperature; C is the surfactant concentration.

.

Table S1. Surface activity parameters of surfactants.

**Surfactant**	**10^{-6} Γ_{cmc} (mol·m^{-2})**	**ΔG_{mic}^{0} (kJ·mol^{-1})**	**ΔG_{ads}^{0} (kJ·mol^{-1})**
**DDPA**	2.35	-26.17	-44.70
**DDBS**	2.51	-27.31	-44.56

Table S2. Compositions and property of Xinjiang crude oil.

Asphaltenes (wt%)	Asphaltenes (wt%)	Aromatics (wt%)	Saturates (wt%)	Wax content (wt%)	Viscosity (mPa·s)	Density ($g·cm^{-3}$)
**0.92**	**6.86**	**12.68**	**69.63**	**5.14**	**15.8**	**0.8614**

Table S3. The contact angle data of water.

Time	contact angle(°)-1	contact angle(°)-2	contact angle(°)-3	Mean	Standard Deviation (SD)	Standard Error(SE)	95% confidence interval(CI)
**0**	111.46	113.79	111.84	112.36	1.25	0.72	112.36±3.11
**2**	111.37	113.32	111.35	112.01	1.13	0.66	112.01±2.82
**4**	111.13	113.15	111.36	111.88	1.11	0.64	111.88±2.75
**5**	111.10	113.11	111.32	111.84	1.11	0.64	111.84±2.75
**6**	111.00	112.91	111.28	111.73	1.03	0.59	111.73±2.56
**8**	110.73	112.80	111.11	111.54	1.10	0.64	111.54±2.74
**10**	110.35	112.46	110.85	111.22	1.10	0.64	111.22±2.74
**12**	110.10	112.13	110.63	110.95	1.05	0.61	110.95±2.62
**14**	109.84	111.86	110.37	110.69	1.05	0.60	110.69±2.60
**15**	109.77	111.72	109.95	110.48	1.08	0.62	110.48±2.68
**16**	109.66	111.60	109.85	110.37	1.07	0.62	110.37±2.66
**18**	109.68	111.31	108.69	109.89	1.33	0.77	109.89±3.29
**20**	109.34	111.11	108.44	109.63	1.36	0.78	109.63±3.37

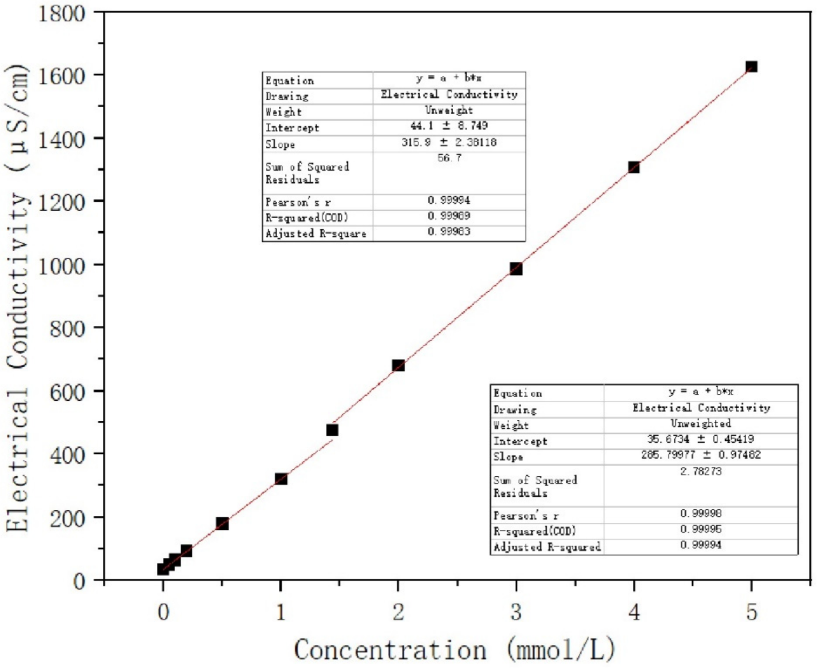

Figure S1. The conductivity-concentration calibration curve of DDPA.

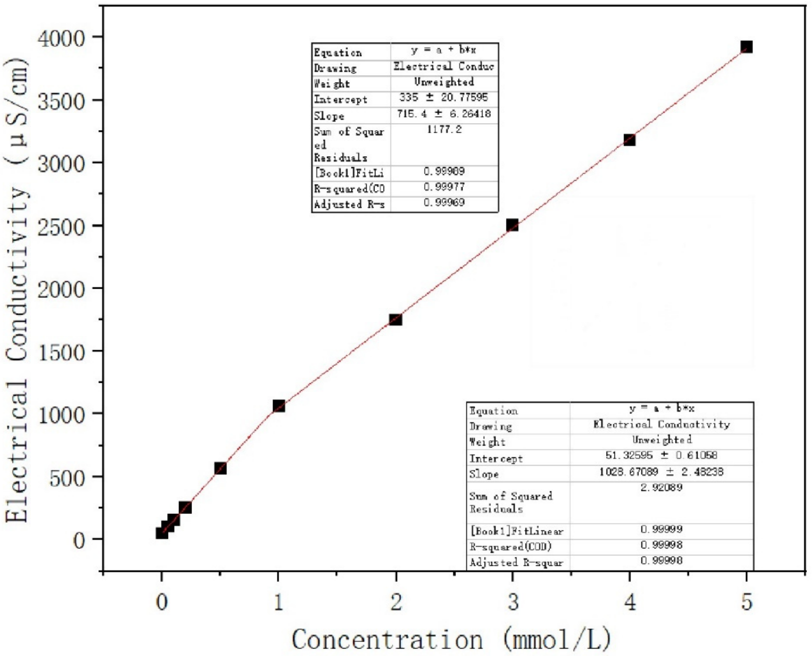

Figure S2. The conductivity-concentration calibration curve of DDBS.

Table S4. Basic parameters of sandstone cores.

Core	Length (cm)	Diameter (cm)	Porosity (%)	Water Permeability (10^{-2} μm^2)	Oil Saturation (%)
**DDPA**	9.881	2.508	19.67	123.63	83.5
**DDBS**	9.539	2.530	19.11	111.02	81.1

Section S2. Specific steps of core displacement experiment.

(1) Measure the length L and diameter D of the artificial core with a vernier caliper, and calculate the core volume V.

$$V = (\pi D^2 L)/4 \tag{S4}$$

(2) Place the core in a constant-temperature drying oven, set it to 120°C and dry for 24 h, after the core reaches a constant weight, remove it, weigh and record the core mass M_1.

(3) Place the dried core into a Büchner funnel, add deionized water, and apply vacuum for 4 h until no more bubbles emerge from the core. Remove the core saturated with water, drain it until free of excess water, and weigh the core to obtain mass M_2.

(4) Calculate the pore volume (V_P) and porosity (φ) of the core using the following formula.

$$V_P = (M_1 - M_2)/\rho \tag{S5}$$

$$\varphi = V_P/V \tag{S6}$$

(5) Prepare simulated formation water and various betaine surfactant solutions, then load them into separate water and solution tanks. Saturate the core and place it in a core holder, securing the inlet and outlet. Apply a confining pressure of 8 MPa. Activate the pressure data acquisition system, start the flow pump, and set the flow rate to 0.4 mL·min^{-1} for the simulated formation water. Once the pressure reaches the equilibrium pressure P_0, record the pressure and calculate the core's water permeability (K) using Darcy's law. The specific data for the simulated formation

water are presented in Table S3. The formula is as follows:

$$K = (Q\mu L)/AP_0 \tag{S7}$$

Here, K represents the water permeability; P_0 denotes the water flooding equilibrium pressure; μ is the viscosity of water; L is the length of the core; A is the cross-sectional area of the core; Q is the flow rate of the injected fluid.

(6) Set the temperature in the incubator to 40°C and pump Xinjiang crude oil into the core at a flow rate of 0.4 $mL \cdot min^{-1}$ until pressure stabilization and no water droplets are produced. Then, shut off the flow pump. Record the displaced aqueous phase volume, which represents the core's saturated oil volume. The oil saturation is calculated as the ratio of the core's saturated oil volume to its pore volume. Subsequently, age the oil-saturated core under experimental conditions for 12 h.

(7) Pump simulated formation water into the core displacement apparatus at a flow rate of 0.4 $mL \cdot min^{-1}$, and record the volumes of crude oil and aqueous phase in a small test tube every 0.4 V_P. When the displacement pressure reaches equilibrium and the water cut reaches 98%, close the water tank valve and switch to the surfactant solution tank via a six-way valve, introducing 0.4 V_P of surfactant solution. Then, reopen the water tank valve through the six-way valve to introduce simulated formation water until the displacement pressure is balanced and the water cut is 98% again. Shut off the flow pump, and record the remaining oil after surfactant flooding, which represents the enhanced oil recovery. Concurrently, monitor and record the pressure changes throughout the displacement process.

Table S5. Composition of simulated formation water.

Na^++K^+ ($mg \cdot L^{-1}$)	Mg^{2+} ($mg \cdot L^{-1}$)	Ca^{2+} ($mg \cdot L^{-1}$)	Cl^- ($mg \cdot L^{-1}$)	SO_4^{2-} ($mg \cdot L^{-1}$)	HCO_3^- ($mg \cdot L^{-1}$)	TDS ($mg \cdot L^{-1}$)
**2992.22**	**21.07**	**86.83**	**2735.23**	**3.49**	**3596.11**	**7636.89**
